# CRISPR/Cas9-mediated gene manipulation to create single-amino-acid-substituted and floxed mice with a cloning-free method

**DOI:** 10.1038/srep42244

**Published:** 2017-02-08

**Authors:** Xiaolong Ma, Chao Chen, Jennifer Veevers, XinMin Zhou, Robert S. Ross, Wei Feng, Ju Chen

**Affiliations:** 1 Department of Cardiothoracic Surgery, The Second Xiangya Hospital, Central South University, Changsha, Hunan, China; 2 Department of Medicine-Cardiology, University of California San Diego, 9500 Gilman Drive, Mail Code 0613-C, La Jolla, California 92093-0613, USA; 3 Veterans Administration Healthcare San Diego, 3350 La Jolla Village Drive, San Diego, California 92161, USA

## Abstract

Clustered regulatory interspaced short palindromic repeats (CRISPR)/CRISPR-associated protein 9 (Cas9) technology is a powerful tool to manipulate the genome with extraordinary simplicity and speed. To generate genetically modified animals, CRISPR/Cas9-mediated genome editing is typically accomplished by microinjection of a mixture of Cas9 DNA/mRNA and single-guide RNA (sgRNA) into zygotes. However, sgRNAs used for this approach require manipulation via molecular cloning as well as *in vitro* transcription. Beyond these complexities, most mutants obtained with this traditional approach are genetically mosaic, yielding several types of cells with different genetic mutations. Recently, a growing body of studies has utilized commercially available Cas9 protein together with sgRNA and a targeting construct to introduce desired mutations. Here, we report a cloning-free method to target the mouse genome by pronuclear injection of a commercial Cas9 protein:crRNA:tracrRNA:single-strand oligodeoxynucleotide (ssODN) complex into mouse zygotes. As illustration of this method, we report the successful generation of global gene-knockout, single-amino-acid-substituted, as well as floxed mice that can be used for conditional gene-targeting. These models were produced with high efficiency to generate non-mosaic mutant mice with a high germline transmission rate.

The clustered regulatory interspaced short palindromic repeat (CRISPR)/CRISPR-associated protein 9 (Cas9) system is a powerful and widely used editing tool for the direct manipulation of the mouse genome in zygotes. It has provided an extremely efficient method for the generation of genetically modified mice to elucidate the functions of genes in development and disease^1^,^2^,^3^.

The CRISPR/Cas9 system was initially described as an acquired prokaryotic immune defense system consisting of a Cas9 nuclease and two small RNAs: CRISPR RNA (crRNA), which acts as a guide for gene targeting, and trans-activating crRNA (tracrRNA), which binds to crRNA and forms a ribonucleoprotein complex with Cas9 to direct sequence-specific Cas9 double-stranded (ds) DNA cleavage^4^,^5^,^6^. Given its simplicity, the CRISPR/Cas9 system has been adopted for robust genome editing by engineering the dual-crRNA:tracrRNA as a chimeric single guide RNA (sgRNA)^4^,^7^. Currently, the most commonly used approach for generating mutant mice with the CRISPR/Cas9 system is to microinject Cas9 mRNA and sgRNA, or their expression plasmid into zygotes^8^,^9^,^10^. However, this approach harbors a number of disadvantages^11^. First, sgRNAs used in this approach require manipulation via molecular cloning as well as *in vitro* transcription. Second, most mutants produced with this current method are genetically mosaic, leading to cells carrying a variety of different insertion or deletion (indel) mutations. This in turn complicates germline transmission of the genetic variant, and can also make phenotypic analysis of founder embryos and mice more complex. Furthermore, increasing evidence has demonstrated that methodology using direct delivery of Cas9 protein can be more efficient than the use of Cas9 mRNA in the generation of non-mosaic mutant mice^1^,^2^,^12^.

To overcome the target vector construction and *in vitro* RNA transcription required for sgRNA, a recent study used crRNA and tracrRNA in combination with commercially available Cas9 protein and a regular gene targeting vector, to generate knock-in mice^2^. In our current study we report a method that capitalized on a cloning-free CRISPR/Cas9 system using commercial Cas9 protein combined with chemically synthesized crRNA, tracrRNA, and single-strand oligodeoxynucleotides (ssODNs). This system allowed us to consistently obtain high-efficiency editing of multiple genes in the mouse, and successfully generate non-mosaic mutant mouse models with a variety of editing schemes, including, frame-shift indel mutations, single-amino-acid substitutions, and LoxP-inserted conditional alleles. Taken together, our method is a simple, cloning-free, and highly efficient technique, which accelerates our ability to produce rapid mouse genome editing *in vivo*.

## Results

### Generation of gene-knockout mice

**Figure 1:**
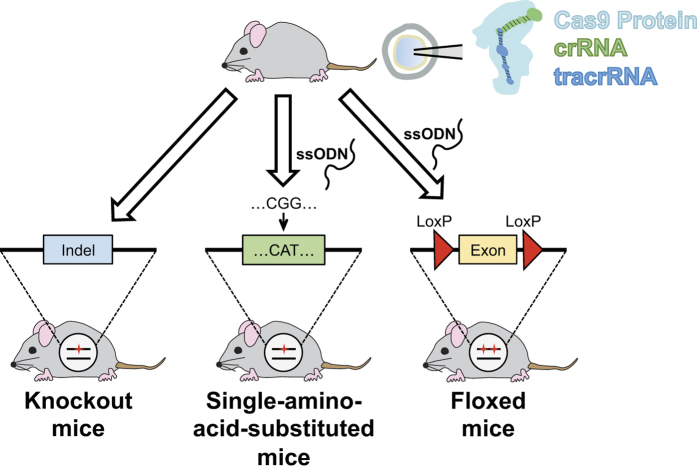
Schematic overview of the study. A cloning-free CRISPR/Cas9 system using commercial Cas9 protein combined with chemically synthesized CRISPR RNA (crRNA), trans-activating crRNA (tracrRNA), with or without single-strand oligodeoxynucleotides (ssODNs) generated non-mosaic mutant mouse models with a variety of editing schemes, including, frame-shift insertion or deletion (indel) mutations, single-amino-acid substitutions, and LoxP-inserted conditional alleles.

**Figure 2:**
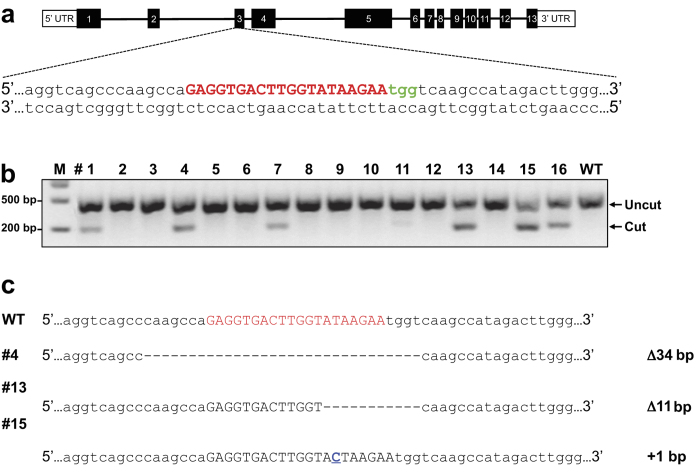
Generation of alpha kinase 2-knockout (ALPK2-KO) mice. **(a)** Sequence of CRISPR RNA (crRNA, red) to target the Cas9 nuclease to a region of exon 3 in the ALPK2 mouse gene. The protospacer adjacent motif (PAM) is shown in green. **(b)** CRISPR/Cas9-mediated genomic modification was revealed by the T7 endonuclease 1 (T7E1) assay. Six of 16 founder mice contained cleavage products. M, molecular marker; WT, wildtype. **(c)** Subsequent sub-cloning of flanking regions surrounding the crRNA targeting site (n = 6 sub-clones/mouse) identified three mice (#4, #13, and #15) with frameshift mutations. In mouse #15, the insertion nucleotide is underlined in blue.

The double-strand breaks (DSBs) induced by the CRISPR/Cas9 system stimulate DNA repair by at least two distinct mechanisms, non-homologous end joining (NHEJ) and homology-directed repair (HDR)^13^,^14^. NHEJ is error-prone and introduces unpredictable patterns of insertions and deletions, which can lead to disruption of the protein-coding capacity of a defined locus. To investigate whether gene-knockout mice can be efficiently generated by direct pronuclear injection of a Cas9 protein:crRNA:tracrRNA complex into mouse zygotes (Fig. 1), we designed crRNA targeting alpha kinase 2 exon 3, which contains the start codon (Fig. 2a). Using the T7 endonuclease 1 (T7E1) assay to detect on-target CRISPR/Cas9 events, we found that six out of 16 (37.5%) mice contained indel mutations (Fig. 2b). Subsequent sub-cloning of flanking regions surrounding the crRNA targeting site identified three mice with frameshift mutations (Fig. 2c). Each of these mice appears to be non-mosaic as they contain only wildtype and one type of mutant allele.

### Generation of single amino-acid-substituted mice

**Figure 3:**
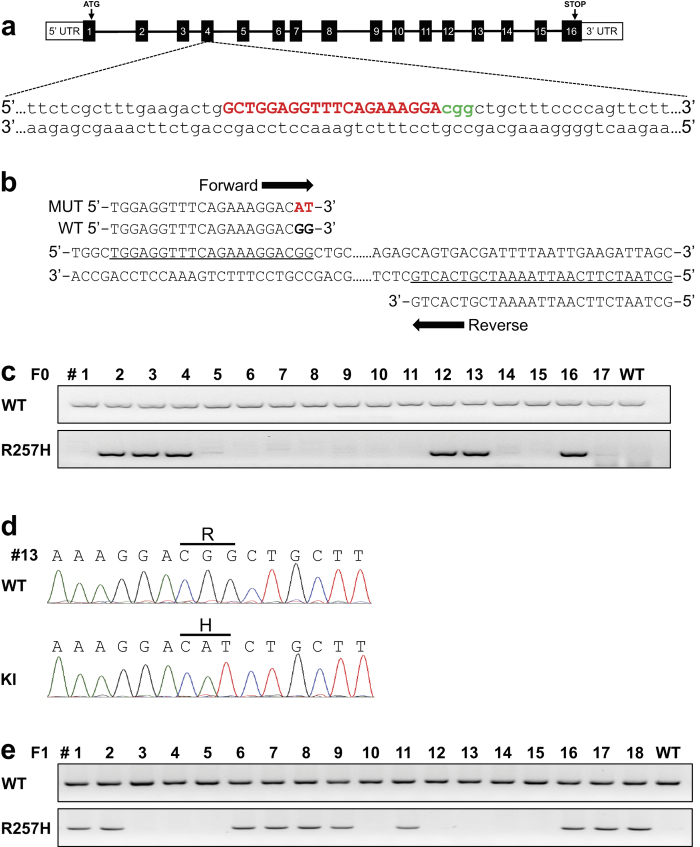
Generation of Nesprin-1α2 R257H knock-in mice. (**a**) Sequence of CRISPR RNA (crRNA, red) to target the Cas9 nuclease to the arginine (R) 257 region in exon 4 in the Nesprin-1α2 (*SYNE1*) mouse gene. The protospacer adjacent motif (PAM) is shown in green. (**b**) Design of the mutation-specific primers to detect the R257 to histidine (H) amino acid substitution. (**c**) PCR screening using mutant-specific primers identified six of 17 newborn mice contained the correct gene variant allele. (**d**) Sub-cloning sequencing shows an individual clone has either wildtype (WT) or R257H knock-in (KI) alleles. A representative sequencing analysis of one founder (#13) of the six PCR-positive mice is shown. (**e**) Germline transmission analysis of the variant alleles to the F1 generation was achieved by backcrossing F0 knock-in mice with wildtype C57/B6 mice. All F0 mice showed successful germline transmission with an average efficiency of 52.8% (ranging from 36% to 71% among different founders, n = 3). A representative genotyping screen of offspring from one founder (#13) is shown.

Single amino-acid substitutions frequently cause genetic diseases in man and thus recapitulation of these variants is used to produce mouse models of disease. To investigate whether mice with single amino-acid-substitutions could be generated easily using the same approach noted above, we aimed to generate founder mice harboring an arginine (257) to histidine amino acid substitution in the Nesprin-1α2 (*SYNE1*) gene (Figs 1 and 3a). We injected a mixture of 30 ng/μL Cas9 protein, 0.6 pmol/μL SYNE1-R257H crRNA, 0.6 pmol/μL tracrRNA, and 20 ng/μL ssODN into the pronuclei of zygotes. In order to quickly screen for correctly targeted mice, we designed mutation-specific primers with a site-specific variant sequence at the 3′ terminus of the forward primer, as shown in Fig. 3b. We found six of 17 newborn mice contained the correct gene variant allele (Fig. 3c). The HDR efficiency of the targeted mutation by Cas9 protein:crRNA:tracrRNA complex injection was thus 35%. The genotypes of PCR-positive mice were confirmed by sequencing the six individual sub-clones (Fig. 3d). We found that the six correctly targeted mice contained only two types of alleles, compared with multiple alleles as reported by Cas9 mRNA/sgRNA injection^15^, indicating that the founders are heterozygous mice rather than chimeras.

To investigate germline transmission of the variant alleles to the F1 generation, we backcrossed F0 knock-in mice with wildtype C57/B6 mice. We tested three different variant lines using PCR and subsequent sequencing, and found that all F0 mice showed successful germline transmission with an average efficiency of 52.8% (ranging from 36% to 71% among different founders). A representative genotyping screen of offspring from one founder (designated #13) is provided (Fig. 3e). Consistent with only two types of alleles in the founders, these results indicate that the founders are heterozygous mice rather than chimeras. We have so far successfully generated eight different variant mice using this method (Table 1).

**Figure 4:**
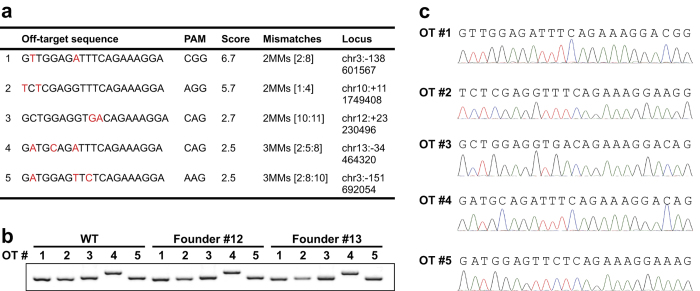
Analysis of off-target candidates at predicted sites in the mouse genome. (**a**) The top five predicted off-target sites for mutagenesis. The mismatch (MM) sequence is labeled in red. PAM, protospacer adjacent motif. **(b)** PCR products flanking each predicted off-target (OT) site (#1–5) in wildtype (WT) and mutant founder (#12 and #13) mice. **(c)** Sub-cloning sequencing (n = 6 sub-clones/mouse) confirmed no off-target cleavage. A representative sequencing analysis of one founder (#13) is shown.

Finally, we investigated off-target cleavage in the CRISPR/Cas9 generated mice produced with our methodology. This has been a serious problem associated with CRISPR/Cas9-mediated genome editing techniques in the past^11^,^16^,^17^. Here we tested the top five predicted off-target sites for mutagenesis using the CRISPR Design (MIT) website^18^ (Fig. 4a). A region flanking each predicted off-target site was amplified by PCR from wildtype and two mutant founders (Fig. 4b). The same molecular size band resulted from all samples, indicating there were no large deletions flanking the sites. Furthermore, we confirmed the sequence flanking the predicted off-target sites by sequencing six individual clones of each founder (Fig. 4c). The sequencing data confirmed no off-target cleavage, suggesting that genome editing by Cas9 protein:crRNA:tracrRNA complex injection is highly specific.

### Generation of conditional knockout mice

**Figure 5:**
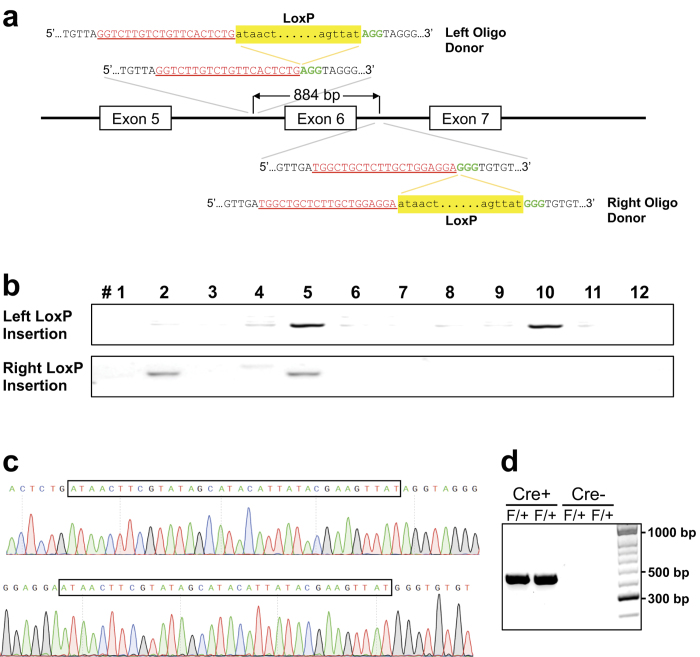
Generation of conditional knockout mice. **(a)** Schematic illustration to generate a conditional allele of the paxillin gene by insertion of two LoxP sites. The sequences of CRISPR RNA (crRNA) to target the Cas9 nuclease to intron 5 (left) and intron 6 (right) are shown in red. The protospacer adjacent motif (PAM) is shown in green. In the oligo donor sequence, the LoxP site is highlighted in yellow. **(b)** PCR screening using mutant-specific primers identified one (#5) of 12 mice had successful insertion of both LoxP sites in the paxillin allele. **(c)** Sub-cloning sequencing confirmed the precise integration of LoxP sites in mouse #5. **(d)** PCR genotyping of paxillin floxed (F) mice crossed with Sox2-Cre mice using primers spanning the two LoxP sites. The presence of an amplified DNA fragment (440 bp) indicates that DNA between the two LoxP sites was deleted by Cre recombinase.

We further investigated whether mice could be generated using this one-step approach by insertion of two LoxP sites into the same allele of the paxillin gene (Fig. 1). Use of this facilitated methodology would greatly accelerate the time to produce a model that could be used to conditionally manipulate a gene of choice. We designed a crRNA targeting the paxillin gene intron 5 (left) and a crRNA targeting paxillin intron 6 (right), as well as the corresponding LoxP site oligos with 60 bp homology sequences on either side surrounding each Cas9-mediated DSB (Fig. 5a). Mutation-specific PCR analysis identified that two out of 12 (17%) mice had either left (#10) or right (#2) LoxP insertion (Fig. 5b). One pup (#5) however, was shown to have successful insertion of both LoxP sites in the paxillin allele (Fig. 5b). The precise integration of LoxP sites into the same allele was confirmed by sequencing PCR products generated from DNA from this model (Fig. 5c). To demonstrate that the floxed allele is indeed functional, we crossed the paxillin floxed mice with germline deleter Cre mice (Sox2-Cre)^19^. As shown in the Fig. 5d, the DNA between the two LoxP sites was deleted by Cre recombinase.

## Discussion

The CRISPR/Cas9 system is proving to be a powerful yet simple tool to manipulate the genome for the generation of genetically modified animals. In this study, we report the successful generation of both single amino-acid-substituted and floxed alleles, using a simplified, cloning-free CRISPR/Cas9-mediated method. Our system offers an advantage over more traditional methods since it generates non-mosaic mutant mouse models, and does so without CRISPR/Cas9 vector construction or *in vitro* RNA transcription manipulation.

In each of our mutant mice lines generated by pronuclear injection of a Cas9 protein:crRNA:tracrRNA complex, with or without ssODN, only two types of alleles resulted with a high frequency of germline transmission (~50%), supporting the notion that founders are germline heterozygous, and not chimeric. In our study, we also did not observe any detectable off-target effects in the top five predicted off-target sites for mutagenesis using Cas9 protein:crRNA:tracrRNA-mediated genome editing, which is consistent with other methods^2^. This suggests that it is feasible to achieve low off-target effects through injection of Cas9 protein into the pro-nuclei of mouse zygotes. No chimerism and detectable off-target effects observed using our approach may reflect that the Cas9 protein has a relatively shorter half-life in embryos than Cas9 mRNA^20^. This phenomenon is also consistent with the finding that a Cas9 protein-RNA complex was rapidly degraded in cultured cells^21^,^22^. The rapid degradation of Cas9 protein may be advantageous in avoiding side effects of Cas9 nuclease activity. Another possibility is that it takes time for Cas9 mRNA to translate into Cas9 protein^23^. When Cas9/sgRNA complexes translocate to the nucleus with the function of the nuclear localization signal, the donor DNA may be transferred in a delayed fashion^24^. In this scenario, repair of DSBs by NHEJ could already be in process prior to the arrival of donor DNA at the target site.

In summary, we report a novel method for the highly efficient generation of non-mosaic mutant mouse models with indel mutations, single-amino-acid substitutions, and LoxP-inserted alleles, through one-step injection of commercially available Cas9 protein combined with chemically synthesized crRNA and tracrRNA. This simple, customizable and ready-to-use genome editing system accelerates our ability to produce rapid mouse genome editing *in vivo.*

## Methods

### CRISPR target sequence design

Guide sequences for CRISPR/Cas9 injection were designed as previously detailed^18^. The target sequence preceding the protospacer adjacent motif (PAM) was obtained from the exon region of the indicated genes.

### Chemical synthesis of crRNA, tracrRNA, and ssODN

The crRNA and tracrRNA were designed as previously reported^2^, chemically synthesized, and RNase-Free HPLC purified by Integrated DNA Technologies (Coralville, IA, USA). Single-strand ODN was chemically synthesized and standard desalted by Integrated DNA Technologies (Coralville, IA, USA). All sequences are listed in Table 2.

### Cas9 proteins

Recombinant Cas9 proteins were obtained from New England BioLabs (Cat #M0386S) (Thousand Oaks, CA, USA).

### Microinjection of zygotes

All animal studies were performed in accordance with the *Guide for the Care and Use of Laboratory Animals*, published by the National Academies Press (US), 2011, 8^th^ Edition, and according to protocols approved by the Institutional Animal Care and Use Committee at the University of California, San Diego. The protocol describing pronuclear microinjection is provided on the University of California, San Diego Transgenic Mouse Core website (https://healthsciences.ucsd.edu/research/moores/shared-resources/transgenic-core/services/Pages/pronuclear-injection.aspx). Briefly, Cas9 proteins, crRNA and tracrRNA, with or without ssODN were diluted and mixed in IDTE buffer (IDT, Coralville, IA, USA) to a working concentration of 30 ng/μL, 0.6 pmol/μL, 0.6 pmol/μL, and 20 ng/μL, respectively. The mixture was incubated at 37 °C for 5 minutes. One picoliter of the mixture was then injected into pronuclei of one-cell stage zygotes from C57BL/6 J mice (The Jackson Laboratory). Based on the published crystal structure, which reveals that Cas9, crRNA, tracrRNA and its DNA target forms a complex at a ratio of 1:1:1:1^25^,^26^, we used equal molecular amounts of each component in the mixture. Based on a previous study^2^, we used 0.6 pmol/μL of crRNA and tracrRNA for microinjection of mouse zygotes. To remain consistent with other reports^27^, we designed our donor DNA to be flanked on each side by ~50 bases homologous to the sequence surrounding the Cas9-mediated DSB.

### PCR screening and sequence analysis

To prepare genomic DNA, mouse tails were incubated in 300 μL of 50 mM NaOH at 98 °C for 30 minutes, then 50 μL of 1 M Tris-HCl (pH 8.0) was added to the resultant solution. Genomic fragments at targets sites were amplified by PCR with Taq (NEB # E5000S) and two pairs of primers. All primers used in this study are listed in Table 3. The PCR conditions were as follows: 30 cycles of 94 °C for 20 seconds, 60 °C for 20 seconds and 72 °C for 30 seconds. The resultant PCR amplicons were analyzed by electrophoresis on a 2% agarose gel. For sequencing, PCR products were further sub-cloned using a Zero Blunt TOPO PCR cloning kit (Life Technologies, Grand Island, NY). The plasmid DNAs containing the genomic fragments were prepared from individual colonies. Six individual clones from each PCR reaction were sequenced.

### T7 endonuclease I (T7E1) assay

The DNA fragment that overlaps the crRNA target site was amplified by PCR using the following primers: forward primer 5′-AGGCTATTCTGTAGCTTCGCTC-3′ and reverse primer 5′-CATTATCTGTCATTCAAGCCGTAAG-3′ The purified PCR product (~250 ng) was denatured and reannealed in NEB Buffer 2 (NEB) to form heteroduplex DNA, which was subsequently digested with T7E1 (NEB, M0302L) at 37 °C for 15 minutes and analyzed on a 2% agarose gel.

### Off-target candidates

Potential off-target sites were predicted using the algorithm determined by the Zhang laboratory (http://crispr.mit.edu/). The 23 bp off-target sequence included the PAM motif of the guide sequence as the algorithm allows more than 23 bp. The top five off-target candidate sites are listed in Fig. 4a.

## Additional Information

**How to cite this article**: Ma, X. *et al*. CRISPR/Cas9-mediated gene manipulation to create single-amino-acid-substituted and floxed mice with a cloning-free method. *Sci. Rep.* **7**, 42244; doi: 10.1038/srep42244 (2017).

**Publisher's note:** Springer Nature remains neutral with regard to jurisdictional claims in published maps and institutional affiliations.

## Figures and Tables

**Table 1:** Generation of mutant mice by Cas9 protein:crRNA:tracrRNA complex injection.

Mouse gene	No. of mice at wean	Amino acid replacement	Mutation sequence	Correct knock-in efficiency
SYNE1	17	R257H	CGG- > CAT	35%
SYNE1	9	W463D464- > AA	TGGGAC- > GCGGCC	33%
SYNE1	15	E646K	GAG- > AAG	53%
NEXN	6	R274C	CGT- > TGT	33%
NEXN	5	G645DEL	GGA- > –	40%
TAX1BP3	14	I33T	ATT- > ACT	50%
FLNC	5	S1625L	TCG- > TTG	60%
TLN	3	L432G	CTT- > GGT	67%

**Table 2:** Sequences of crRNA, tracrRNA, and single-strand oligodeoxynucleotides used in this study.

Oligo name	Sequence
R257H-crRNA	5′-gcuggagguuucagaaaggaGUUUUAGAGCUAUGCUGUUUUG-3′
tracrRNA	5′-AAACAGCAUAGCAAGUUAAAAUAAGGCUAGUCCGUUAUCAAC UUGAAAAAGUGGCACCGAGUCGGUGCU-3′
R257H-DNA	5′-GCAGAAGTTTCTGGATGACTATTCTCGCTTTGAAGACTGGCTGGA GGTTTCAGAAAGGACATCTGCTTTCCCCAGTTCTTCCGGGGTGCTCT ATACAGTTGCCAAGGAGGAGCTGAAGAAG-3′
ALPK2-crRNA	5′-gaggugacuugguauaagaaGUUUUAGAGCUAUGCUGUUUUG-3′
PXN-L-crRNA	5′-ggucuugucuguucacucugGUUUUAGAGCUAUGCUGUUUUG-3′
PXN-R-crRNA	5′-uggcugcucuugcuggaggaGUUUUAGAGCUAUGCUGUUUUG-3′
PXN-L-DNA	5′-TTCATAGCTCTGCTGCTCACAGGATGCTCCTAGTAATGTTAGGTCTTG TCTGTTCACTCTGATAACTTCGTATAGCATACATTATACGAAGTTATAGGT AGGGTCTCCCTCCACAGCCCGGACTGTTGTGGAACTCACTACGTAGTAA GCCTCTTC-3′
PXN-R-DNA	5′-ATCAGGAGGCACGGACACAGGGACAAAGAGCCCTGTGTTGATGGCTGCT CTTGCTGGAGGAATAACTTCGTATAGCATACATTATACGAAGTTATGGGTGT GTCCTGCTGGGCCCGTGGGATCAGGATCCCAAGCCTGAGAGGTCACTTGCC CCAG-3′

ALPK, alpha kinase 2; PXN, paxillin.

**Table 3:** Primers used in this study.

Primer name	Sequence	GC%	Tm
ALPK2-Forward(seq)	TTAGCATATCCCATTGTGCTCATC	42	62
ALPK2-Reverse(seq)	AATTACTAGTGTGCTATACTGTCTAG	35	61.7
SYNE1-R257H-F(wt)	TGGAGGTTTCAGAAAGGACGG	52	61.2
SYNE1-R257H-F(mut)	TGGAGGTTTCAGAAAGGACAT	43	57.5
SYNE1-R257H-F(rev)	GCTAATCTTCAATTAAAATCGTCACTG	33	62.1
SYNE1-R257H-F(seq)	TCCCTGGGCGGTTTGGGTC	68	63.6
OT1-Forward(seq)	GCTGAGTGCGACTCCGTCC	68	63.6
OT1-Reverse(seq)	GGCTAGGTGATAAGGCTCTAGC	55	64.2
OT2-Forward(seq)	GTCTTTGGTCTATGTGTGATCATG	42	62
OT2-Reverse(seq)	CAAAGACAGGTGCAGAACAGG	52	61.2
OT3-Forward(seq)	GTGTAAGACATTGTGTGAAGTCCT	42	62
OT3-Reverse(seq)	CTGTTGCTCACAGAGTCGCCA	57	63.2
OT4-Forward(seq)	ATGGCATTGAAACCTTGTCAGCATA	40	62.5
OT4-Reverse(seq)	ACCAGCCAGTGTGTAACCAGC	57	63.2
OT5-Forward(seq)	GACAGTCACCGACTTGTAAAGC	50	62.1
OT5-Reverse(seq)	CCCTGCCACTGTGCTATCATG	57	63.2
PXN-L-Forward(mut)	AACGTGGTCACGAGCCCAGC	65	64.6
PXN-L-Reverse(mut)	GACCCTACCTATAACTTCGTATAATG	38	62.9
PXN-R-Forward(mut)	CATTCTTCGACCTCAGTTGAGTAG	46	63.6
PXN-R-Reverse(mut)	GACACACCCATAACTTCGTATAAT	38	60.3
PXN-Forward(seq)	TCACGGGATGAGACGACATAGG	55	64.2
PXN-Reverse(seq)	GTGTTCTGAGGACAGCAAGCG	57	63.2
PXN-CRE-Forward	TCCAGTGAGACCATGTAGTCCC	55	64
PXN-CRE-Reverse	GCCCTATCAAGGCAATACATCTTC	46	63.6

ALPK, alpha kinase 2; SYNE1, nesprin-1α2; OT, off-target; PXN, paxillin, Tm, melting temperature.
